# Role of Secreted Conjunctival Mucosal Cytokine and Chemokine Proteins in Different Stages of Trachomatous Disease

**DOI:** 10.1371/journal.pntd.0000264

**Published:** 2008-07-16

**Authors:** Troy A. Skwor, Berna Atik, Raj Prasad Kandel, Him Kant Adhikari, Bassant Sharma, Deborah Dean

**Affiliations:** 1 Center for Immunobiology and Vaccine Development, Children's Hospital Oakland Research Institute, Oakland, California, United States of America; 2 Lumbini Rana-Ambika Eye Hospital, Bhairahawa, Nepal; 3 Department of Medicine, University of California at San Francisco, San Francisco, California, United States of America; 4 UCSF and UCB Joint Graduate Group in Bioengineering, University of California at San Francisco, San Francisco, California, United States of America, and University of California at Berkeley, Berkeley, California, United States of America; Weill Medical College of Cornell University, United States of America

## Abstract

**Background:**

*Chlamydia trachomatis* is responsible for trachoma, the primary cause of preventable blindness worldwide. Plans to eradicate trachoma using the World Health Organization's SAFE program (Surgery, Antibiotics, Facial Cleanliness and Environment Improvement) have resulted in recurrence of infection and disease following cessation of treatment in many endemic countries, suggesting the need for a vaccine to control infection and trachomatous disease. Vaccine development requires, in part, knowledge of the mucosal host immune responses in both healthy and trachomatous conjuctivae—an area of research that remains insufficiently studied.

**Methodology/Principal Findings:**

We characterized 25 secreted cytokines and chemokines from the conjunctival mucosa of individuals residing in a trachoma endemic region of Nepal using Luminex X100 multiplexing technology. Immunomodulating effects of concurrent *C. trachomatis* infection were also examined. We found that proinflammatory cytokines IL-1β (r = 0.259, *P* = 0.001) and TNFα (r = 0.168, *P*<0.05) were significantly associated with trachomatous disease and concurrent *C. trachomatis* infection compared with age and sex matched controls from the same region who did not have trachoma. In support of these findings, anti-inflammatory cytokine IL-1 receptor antagonist (IL-1Ra) was negatively associated with chronic scarring trachoma (r = −0.249, *P* = 0.001). Additional cytokines (Th1, IL-12p40 [r = −0.212, *P*<0.01], and Th2, IL-4 and IL-13 [r = −0.165 and −0.189, respectively, *P*<0.05 for both]) were negatively associated with chronic scarring trachoma, suggesting a protective role. Conversely, a pathogenic role for the Th3/Tr1 cytokine IL-10 (r = 0.180, *P*<0.05) was evident with increased levels for all trachoma grades. New risk factors for chronic scarring trachoma included IL-6 and IL-15 (r = 0.259 and 0.292, respectively, *P*<0.005 for both) with increased levels for concurrent *C. trachomatis* infections (r = 0.206, *P*<0.05, and r = 0.304, *P*<0.005, respectively). Chemokine protein levels for CCL11 (Eotaxin), CXCL8 (IL-8), CXCL9 (MIG), and CCL2 (MCP-1) were elevated in chronic scarring trachoma compared with age and sex matched controls (*P*<0.05, for all).

**Conclusions/Significance:**

Our quantitative detection of previously uncharacterized and partially characterized cytokines, a soluble cytokine receptor, and chemokines for each trachoma grade and associations with *C. trachomatis* infections provide, to date, the most comprehensive immunologic evaluation of trachoma. These findings highlight novel pathologic and protective factors involved in trachomatous disease, which will aid in designing immunomodulating therapeutics and a vaccine.

## Author Summary

Trachoma, a disease of antiquity dating back to the 16th century B.C.E., predominates among developing countries, where it remains the primary cause of preventable blindness worldwide. In trachoma, recurrent *Chlamydia trachomatis* bacterial infections during childhood are thought to result in inflammation and subsequent conjunctival scarring that can progress to trichiasis (TT; chronic trachoma; inversion of ≥1 eyelash that touches the globe of the eye). The trachomatous follicular grade (TF; active disease) is a self-limiting disease, suggesting the coexistence of protective inflammatory proteins. The trachomatous inflammatory grade (TI; active disease) is more likely to progress to trachomatous scarring (TS; chronic trachoma). To date, there are only a handful of studies that have examined the immune response in trachoma, and these were primarily based on gene expression. Characterizing quantified conjunctival mucosal immune differences for secreted proteins among individuals with no, active, and chronic trachoma may identify protein biomarkers associated with protection versus disease, which would greatly aid our understanding of the immunopathogenesis of trachoma. In this study, we characterized 25 cytokine and chemokine proteins for all trachoma grades. We identified eight cytokines and chemokines as risk factors for chronic trachoma and four as protective. Together, these findings further characterize the immunopathologic responses involved during trachoma, which will likely aid in the design of a vaccine and immunomodulating therapeutics for trachoma.

## Introduction

Trachoma, the leading global cause of preventable blindness, has plagued populations for thousands of years [1]. There are over 360 million trachoma cases of whom ∼6 million are blind [2],[3]. Limited access to clean water and inadequate sanitary conditions provides ideal conditions for the persistence of this endemic disease caused by the obligate intracellular bacterium *Chlamydia trachomatis*. Additionally, *C. trachomatis* is the leading bacterial cause of sexually transmitted diseases (STD) throughout the world [4], causing chronic conditions such as arthritis [5], infertility and ectopic pregnancy [6]. The economics and health burden from trachoma alone claims billions of dollars in productivity loss in already poverty stricken countries [7]. Recognizing this, the World Health Organization (WHO) proposed the SAFE program (Surgery, Antibiotics, Facial cleanliness and Environmental improvement) in 2001 with the goal of eradicating blinding trachoma by the year 2020.

In trachoma endemic villages, disease progression is associated with repeated *C. trachomatis* infections of the conjunctivae, which can eventually lead to scarring and blindness from trachomatous trichiasis (TT; ≥1 in-turned eyelash) [1]. Recently, we and others have demonstrated that the S and A components of the SAFE program results in recurrent *C. trachomatis* infections and disease in endemic countries 6–24 months following cessation of treatment [8],[9],[10],[11],[12],[13]. Additionally, the difficulty in implementing and sustaining the F and E programs in resource poor countries suggests that a vaccine is the most effective method of control.

Designing efficacious vaccine candidates requires, in part, characterization of host inflammatory risk factors versus protective immunological components that are associated with the different grades of trachoma. These include follicular trachomatous inflammation (TF); intense trachomatous inflammation (TI); trachomatous conjunctival scarring (TS), trachomatous trichiasis (TT) and corneal opacity (CO) [14]. A limited number of animal models and human studies have begun to elucidate the host immune response in trachoma. The use of Giemsa stains and immunohistochemistry has characterized active trachoma (TF, TI or both) in young children as an epithelial and stromal infiltrate containing neutrophils [15], macrophages, dendritic cells, and B and T lymphocytes [16]. Physiologic characterization has been obtained from non-human primate models where trachoma-induced immunopathology consisted of conjunctival follicles containing B cells coexisting with a rich T lymphocyte population in the perifollicular area [17],[18]. These T lymphocytes were associated with macrophages, and epithelial and stromal cells. The quantity and size of trachoma-related follicles have been shown to directly correlate with the frequency of repeated *C. trachomatis* infections in the non-human primate model [17].

Characterization of intense inflammation during early stages of trachomatous disease exhibits elevated transcriptional levels of the proinflammatory cytokines TNFα and IL-1β, and amplified expression in the presence of *C. trachomatis* infection in human populations [19],[20],[21]. These studies also linked elevated Th1 cytokines IL-12 and IFNγ mRNA levels in conjunctival samples with TI cases [19],[20],[21],[22] and diminished levels in the presence of scarring [19]. No mRNA expression was detected for the Th2 cytokines IL-4 and IL-5 for any trachoma grade [19],[21]. However, elevated IL-10 mRNA levels were apparent in TF and TI cases who were also infected with *C. trachomatis* [21],[22]. Together these data suggest Th1 polarization as protective and IL-10 as a risk factor for exacerbating disease progression.

As trachomatous scarring develops, there is a diminished lymphocytic proliferative response to *C. trachomatis* elementary bodies (EBs) in both cynologus monkeys [23] and humans [24],[25]. Significantly elevated levels of T cytotoxic/suppressor cells compared to helper T lymphocytes have been shown in monkeys with chronic ocular infections [17],[23],[26]. Similarly, this immunosuppressive polarized environment was mirrored in infected TS cases where conjunctival IFNγ, TNFα, and IL-12 mRNA expression levels were decreased compared to levels among TF/TI cases, although IL-1β and TGFβ1 levels were consistently elevated for all grades of trachoma [19]. However, these studies have not examined the post-transcriptional and –translational regulation of these cytokines and their respective protein levels, making it difficult to assess their relevance to trachoma [27],[28],[29],[30]. To date, only one study has evaluated protein levels in which TS cases were shown to have significantly elevated TNFα levels compared with controls [31].

While the above data have started to elucidate our understanding of the immune responses in trachoma, the lack of cytokine protein data prevent us from determining the immunological modulators associated with successive disease severity and identifying protective versus pathologic risk factors. Furthermore, resolving infection and disease relies on localizing and attracting effective leukocyte populations to the infected site. Chemokines are a group of small molecular weight proteins (8–12 kDa) responsible for such a task. However, there have been no studies of chemokines and their association with trachoma. To begin to address these deficiencies, we quantitatively analyzed 25 secreted conjunctival mucosal cytokine and chemokine proteins in a trachoma endemic Nepali population. We detected previously uncharacterized and partially characterized cytokines, a soluble cytokine receptor, and chemokines for each grade of trachoma, and the effect of *C. trachomatis* infections on their production. Our data provide the most comprehensive immunological evaluation associated with trachoma to date.

## Methods

### Study population, trachoma grading, and ethics

This cross-sectional study included 208 individuals residing in a trachoma endemic region of Southwestern Nepal that were age and sex matched post hoc using a stratified sampling method. Samples from mutually exclusive trachoma grades (TF/TI, TS, TT, and Normal) were placed into separate strata by age (5 year spread) and sex. Samples were randomly selected (using a table of random numbers) from each age and gender stratum for each trachoma grade and matched with randomly selected samples from each age and gender stratum for normals. Verbal informed consent was obtained from all study subjects following the institutional review board approval by Children's Hospital and Research Center at Oakland, CA, and the Nepal Netra Jhoti Shang. Verbal consent was documented on an information sheet by the team member who consented each individual who agreed to be a study subject. Grading of the upper tarsus of each study subject was performed according to a modified grading scale previously described by WHO [14]. Briefly, the grades were: no evidence of trachoma characterized by ≤4 follicles on the lower 2/3 of the upper tarsal conjunctiva (Normal denoted as T0), follicular and/or intense trachomatous inflammation (TF/TI), trachomatous conjunctival scarring (TS), trachomatous trichiasis (TT) and TT with inflammation (TT/TI). An agreement between two of three independent readers (authors DD and RK, and Dr. Tracey Hessel) on final grading was made for each patient.

### Sample collection

Conjunctival mucosal secretions were obtained by applying a sterile Weck-cel sponge (Medtronic Inc., Minneapolis, MN) to the inner canthus of each eye and allowing the swab to reach saturation. Samples were placed in a sterile eppendorf tube. A Dacron swab (Remel Inc., Lenexa, KS) was used to sample the upper tarsal and lower conjunctivae of each eye and was placed in M4-RT media (Micro Test Inc., Lilburn, GA) immediately following collection. All swabs were kept on ice for no longer than eight hours until transfer to liquid nitrogen tanks and then to Children's Hospital Oakland Research Institute, Oakland, CA and stored at −80°C until analyzed.

### Detection of *C. trachomatis* in conjunctival samples

DNA was isolated from a M4-RT media containing the conjunctival swabs as previously described [32] following manufacturer's instructions. Samples were defined as positive at an OD_450 nm_>0.8 and negative at an OD_450 nm_<0.2 [32]. Equivocal samples were defined as those that fell within an OD_450 nm_ of 0.2 to 0.8, and were further evaluated using an in-house validation PCR test to assess the presence or absence of chlamydiae as we have described previously [10]. Briefly, DNA from equivocal and negative and positive control samples were amplified using primers flanking the *ompA* gene. The presence of a 1200 bp band corresponding to the positive *C. trachomatis* DNA control, while the negative control was negative, defined an equivocal sample as positive for *C. trachomatis*.

### Detection of cytokine and chemokine protein levels in conjunctival mucosal samples

Mucosal sponges were thawed on ice, and 80 µl of resuspension fluid [50 mM Tris, 0.15 M NaCl, 10 mM CaCl_2_, serine and cysteine protease inhibitors (Protease Inhibitor Cocktail tablets, Roche Diagnostics, Mannheim, Germany) at pH 7.5] was applied to each sponge. Fluid was extracted, and insoluble protein separated via centrifugation at 10,000×g for 10 min at 4°C.

Samples were applied to a Human Cytokine/Chemokine 25-plex [IL-1β, IL-2, IL-4, IL-5, IL-6, IL-7, IL-8 (CXCL8), IL-10, IL-12p40, IL-13, IL-15, IL-17, IFNα, IFNγ, TNFα, GM-CSF, CCL2 (MCP-1), CCL3 (MIP-1α), CCL4 (MIP-1β), CCL11 (Eotaxin), CXCL10 (IP-10), CXCL9 (MIG), CCL5 (RANTES), IL-1Ra, and IL-2R] 96 well plate assay (Biosource International, Inc., Camarillo, CA) following manufacturer's instructions. Briefly, 25-Plex beads were vortexed and sonicated to disperse aggregates, and washed using a vacuum manifold not exceeding 5 psi. Subsequently, 25-Plex beads were incubated with 50 µl of sample or standards for 2 h on an orbital shaker at 500 rpm. Wells were aspirated and washed as described above through the vacuum manifold. Biotinylated detector antibodies were added and incubated on the orbital shaker for 1 h with subsequent washes. After the addition of Streptavidin-RPE, the plates were analyzed using a Luminex X100 instrument (Luminex Technologies, Inc) to determine the quantities of each protein. Calibration was performed before each run, and all 25 cytokines/chemokines were gated (9050–12050) to eliminate bead aggregates and debris associated with the samples. Luminex analysis was set to 50 µl/sample and 80 events/bead. Standard curves were assessed from duplicates consisting of all 25 cytokines/chemokines using a five parameter logistic modeling system. Contiguous dilutions (8 three-fold followed by 3 two-fold dilutions) were applied to the standards, and resuspension fluid was used to determine background. Sensitivity levels were determined for each cytokine/chemokine as the lowest dilution, two standard deviations above background. Sensitivity levels were as follows: TNFα (0.2 pg/ml), IL-1β (1.9 pg/ml), IL-1Ra (14–31560 pg/ml), IL-6 (2.6 pg/ml), IL-4 (1.0 pg/ml), IL-5 (0.8 pg/ml), IL-13 (2.3 pg/ml), IL-12p40 (2.2 pg/ml), IFNγ (0.9 pg/ml), IL-10 (0.3 pg/ml), IL-2 (0.9 pg/ml), IL-2R (4.5 pg/ml), IL-15 (2.1 pg/ml), IFNα (4.2 pg/ml), IL-7 (16.2 pg/ml), IL-17 (5.6 pg/ml), Eotaxin (0.3 pg/ml), GM-CSF (2.7 pg/ml), IL-8 (2.7 pg/ml), MCP-1 (12.0 pg/ml), MIG (9.9 pg/ml), IP-10 (4.0 pg/ml), MIP-1α (4.3 pg/ml), MIP-1β (5.5 pg/ml) and RANTES (8.9 pg/ml).

### Statistical analysis

**Figure 1:**
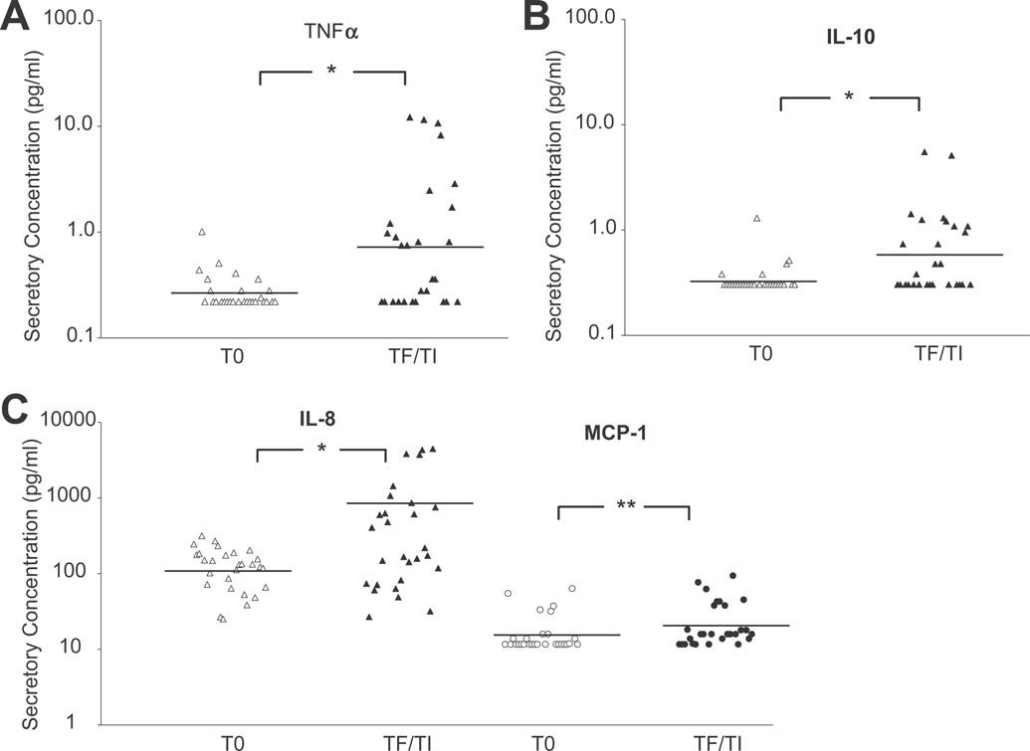
Elevated concentrations of TNFα, IL-10, IL-8 and MCP-1 predominated in TF/TI conjunctival mucosal secretions. Concentrations of conjunctival mucosal cytokines and chemokines among individuals residing in a trachoma endemic region of Nepal were analyzed using multiplexing Luminex technology (see methods). The data are grouped by three categories: (A) proinflammatory cytokines, (B) Th1/Th2/Th3 cytokines and (C) chemokines, representing individual conjunctival mucosal secretory concentrations from TF/TI cases (*n* = 28) and age and sex matched controls (T0, *n* = 28). Lines designate the geometric means of each group. Statistical differences for cytokine and chemokine concentrations between cases with TF/TI and controls with T0 were denoted by * *P*<0.05 or ** *P*<0.01, Wilcoxon rank-sum test.

We used a stratified sampling method to divide the population into mutually exclusive groups based on disease grade (TF/TI vs T0, TS vs T0, etc) as described above. Cytokine and chemokine data, comparing particular disease grades versus age and sex matched controls, were assessed for normality by Shapiro-Wilk W test to determine the appropriate statistical tests. Two-sample Wilcoxon rank-sum (Mann-Whitney) test was performed when p values were <0.05 from the Shapiro-Wilk test. Student t test with equal variance was used when p values were >0.05 (Figures 1–4). Significance for the association between cytokine/chemokine production and different trachoma grades was determined using multiple logistic regression and adjusting for age and *C. trachomatis* infection for the TF/TI cases. Statistical analysis of immuno-modulating effects by *C. trachomatis* for trachoma cases or controls was determined using frequency of cytokine or chemokine detection as the dependent variable adjusting for age. We used Spearman's rank test to quantify the association between individual cytokines/chemokines and different trachoma grades and infection. All statistics were performed using Stata 9.0 software (Stata Corp, College Station, TX).

**Figure 2 pntd-0000264-g002:**
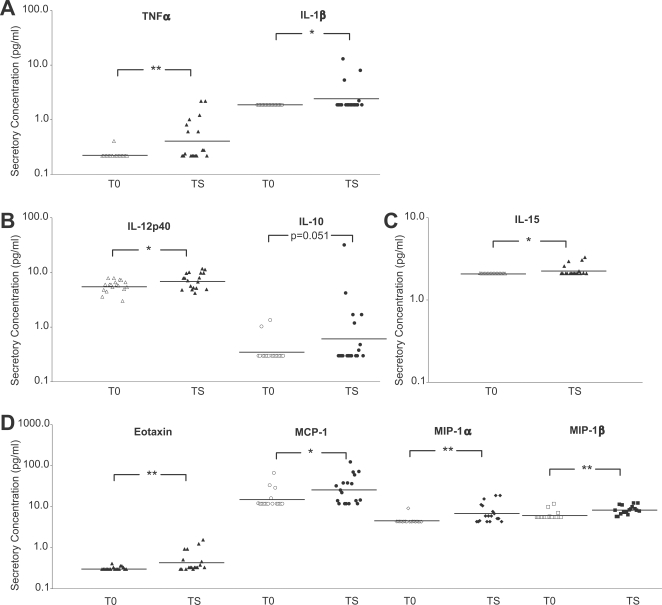
Multiple inflammatory mediators are elevated among individuals with trachomatous scarring (TS) compared to controls. Cytokine and chemokine concentrations within conjunctival mucosal secretions from Nepali patients exhibiting TS were compared to age and sex matched controls. The data are grouped into significant difference within four categories: (A) proinflammatory cytokines, (B) Th1/Th2/Th3 cytokines, (C) IL-2 cytokine family, and (D) chemokines, representing individual conjunctival mucosal secretory concentrations from TS patients (*n* = 18) and age and sex matched controls (T0, *n* = 18). Lines designate geometric means of each group. Statistical differences for cytokine and chemokine concentrations between TS and T0 was denoted * *P*<0.05, ** *P*<0.01 and *** *P*<0.001, Wilcoxon rank-sum test or Student t test when Shapiro-Wilk W test ≥0.05.

**Figure 3 pntd-0000264-g003:**
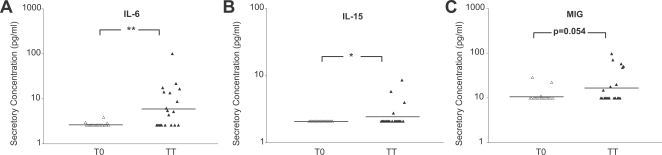
Trichiasis patients have elevated IL-6 and IL-15 levels compared to controls. Cytokine and chemokine concentrations within conjunctival mucosal secretions from Nepali patients exhibiting TT were compared to age and sex matched controls. The data are grouped into significant difference within three categories: (A) proinflammatory cytokines, (B) IL-2 cytokine family and (C) chemokines, representing individual conjunctival mucosal secretory concentrations from TT patients (*n* = 20) and age and sex matched controls (T0, *n* = 20). Lines designate geometric means of each group. Statistical differences between TT and TO was denoted * *P*<0.05 or ** *P*<0.01, Wilcoxon rank-sum test.

**Figure 4:**
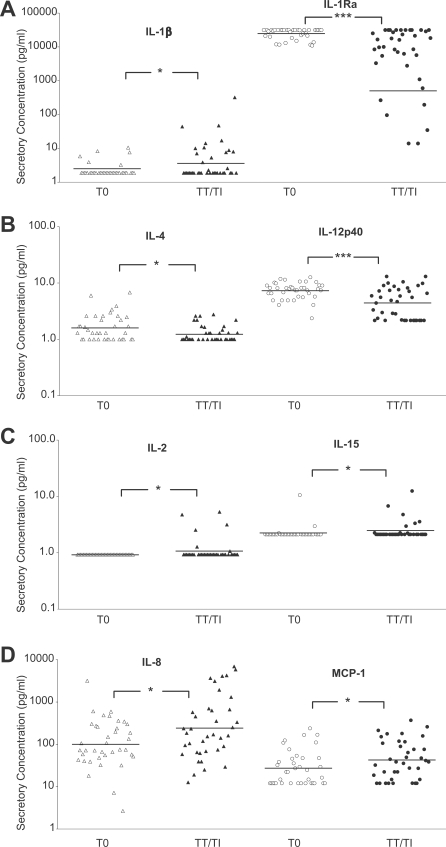
Persistent IL-1β elevation with significantly diminished IL-1Ra and IL-12p40 levels distinguish TT/TI from controls. Cytokine and chemokine concentrations within conjunctival mucosal secretions from Nepali patients exhibiting TT/TI were compared to age and sex matched controls. The data are grouped into four categories with significant differences: (A) proinflammatory cytokines, (B) Th1/Th2/Th3, (C) IL-2 cytokine family, and (D) chemokines, representing individual conjunctival mucosal secretory concentrations from TT/TI patients (*n* = 38) and age and sex matched controls (T0, *n* = 38). Lines designate geometric means of each group. Statistical differences between TT/TI and T0 was denoted * *P*<0.05, ** *P*<0.01 and *** *P*<0.001, Wilcoxon rank-sum test.

## Results

### Clinical and microbiologic characteristics of the study population

Our Nepali study population comprised individuals with TF/TI, TS, TT, and TT/TI who were age and sex matched with identical numbers of individuals without disease (T0) from the same community (Table 1). We assessed the prevalence of *C. trachomatis* infection with different grades of trachoma. Table 1 shows that there was an inverse association between *C. trachomatis* infection and age [*P* = 0.003; OR = 0.97 (0.95–0.99)] while infection was directly associated with TF/TI cases [(*P* = 0.017; OR = 4.4 (1.30–14.92)]. The presence of chronic trachoma defined as any grade demonstrating scarring (TS, TT and TT/TI) was associated with age [*P*<0.001; OR = 1.20 (1.12–1.30)].

**Table 1:** Characteristics of the Nepali population by age, gender, trachoma grades and *C. trachomatis* infection.

	Grade of Disease								Total
	T0	TI	T0	TS	T0	TT	T0	TT/TI	
Age in years [Median (range)]^A^^,^ B	16.5 (3–40)	16.9 (5–40)	37.5 (16–70)	39.7 (16–70)	46.4 (16–70)	48.9 (18–75)	40.0 (13–75)	42.6 (13–75)	35.4
Male	12	12	8	8	9	9	14	14	86
Female	16	16	10	10	11	11	24	24	122
*C. trachomatis* infection	5/27	14/28^c^	4/18	3/18	0/20	5/20	2/37	5/38	38/208

A Age was significantly associated with chronic trachoma (TS, TT, TT/TI) [*P*<0.001, OR = 1.20 (95% CI, 1.12–1.30)].

B Age was inversely significantly associated with *C. trachomatis* infection [*P* = 0.003; OR = 0.97 (95% CI, 0.95–0.99)].

C Significant association of TI with active *C. trachomatis* infection compared to age and sex matched controls with T0 [*P* = 0.017; OR = 4.4 (95% CI, 1.30–14.92)].

### Pro-inflammatory mediators in conjunctival mucosal secretions are associated with trachomatous inflammation

We found significantly elevated protein concentrations of the pro-inflammatory cytokine TNFα for TF/TI cases compared to age and sex matched controls (T0) (Figure 1A, *P* = 0.003) but not for the pro-inflammatory cytokine IL-1β. For the pleiotropic cytokine IL-6, which has been linked to acute and chronic inflammation [33], we found a greater percentage of TF/TI cases (50%) that produced IL-6 compared with controls (25%) (Table S1).

A previous study suggested a protective effect with elevated Th1, IL-12 and IFNγ, mRNA expression for cases with TF/TI, but not for those with scarring [19]. At the protein level, we did not find the same association for TF/TI cases. For Th2 cytokines, we detected IL-4 and IL13 protein levels in over 60% of TF/TI cases compared to controls. However, minimal differences in protein concentrations were observed between the two groups (data not shown). The anti-inflammatory and Th2 cytokine IL-10, which is also referred to as a Th3/T regulatory cytokine 1 (Tr1), was significantly associated with TF/TI cases (Figure 1B, *P* = 0.005) where the mean represented a three-fold higher concentration compared with controls.

We found no significant elevation in any of the IL-2 family of cytokines and receptors (IL-2, IL-7, IL-15, and IL-2R) for the TF/TI cases (Table S1 and data not shown).

We found minimal differences in conjunctival mucosal chemokine protein levels among TF/TI cases except for the CXC family where significantly elevated levels of IL-8 and MCP-1 were found among TF/TI cases compared to controls (Figure 1C; *P*<0.05 and *P*<0.005, respectively). These chemokines are primarily associated with attracting and activating neutrophils, monocytes and T lymphocytes [34], which have all been linked to the immunopathology associated with TF/TI [15],[16].

### Elevated pro-inflammatory cytokine and chemokine proteins in conjunctival mucosal secretions predominate in trachomatous scarring cases

Cases with scarring had significantly elevated levels of the proinflammatory cytokines TNFα and IL-1β compared to controls (Figure 2A, *P* = 0.001 and *P*<0.05, respectively). Levels of IL-6 were barely detected among these cases. However, there was an overall greater frequency of detectable IL-6 protein levels among TS cases (Table S1, 17% TS vs 0% T0), which was similar to the TF/TI cases mentioned above.

The Th2 cytokine IL-13 was decreased in TS cases compared with controls (Table S1, *P*<0.005), while IL-12p40 was elevated (Figure 2B, *P*<0.05), suggesting a slight polarization towards the Th1 phenotype. Elevation in the Th3/Tr1 cytokine IL-10 protein levels for TS cases, although not quite significant (Figure 2B, *P* = 0.051), suggested immuno-regulatory patterns coexisting with Th1. A distinguishing finding for TS cases was the significant protein elevation of an IL-2 family cytokine, IL-15 (Figure 2C, *P*<0.05). This cytokine is produced by a plethora of cell types, including epithelial, monocytic and dendritic cells, and is involved in the activation of effector memory T lymphocytes [35].

Significantly elevated levels of Th1 CC chemokines, MIP-1α and MIP-1β (Figure 2D, *P*<0.01 and *P*<0.001 respectively), and Th2 chemokines, MCP-1 and Eotaxin (Figure 2D, *P*<0.01) were found for the TS cases compared with controls.

### Elevated IL-6 protein levels in conjunctival mucosal secretions predominate among trachomatous trichiasis cases

Conjunctival mucosal secretions demonstrated minimal differences at the protein level among proinflammatory and Th1, Th2 and Th3 cytokines for TT cases (data not shown). However, IL-6 protein levels were significantly elevated among cases with TT compared to controls (Figure 3A, *P*<0.01) as in the other trachoma grades. For the IL-2 family of cytokines, minimal differences were observed except for significantly elevated concentrations of IL-15 (Figure 3B, *P*<0.05), which further suggests that this cytokine is a risk factor for chronic trachoma.

For chemokines, TT cases had increased MIG protein levels compared with controls (Figure 3C, *P* = 0.05), which were not observed in other trachoma grades.

### Low levels of conjunctival mucosal IL-12p40 and IL-1Ra distinguish trachomatous trichiasis with inflammation cases from controls

To our knowledge, the inflammatory response in TT/TI cases has been characterized only once for a limited number of cytokine mRNA expression levels [19]. In our study, the most novel finding was the significant reduction in IL-1Ra protein for cases with TT/TI (Figure 4A, *P*<0.001) compared to controls, further supporting a chronic inflammatory environment for this grade. This is the first time an IL-1β antagonist has been characterized in trachomatous disease. In agreement with an inflammatory environment, significantly higher protein levels of IL-1β were also evident in TT/TI cases compared to controls (Figure 4A, *P*<0.05).

For Th1, Th2 and Th3 cytokines, TT/TI cases had significantly reduced protein levels for the Th1 cytokine IL-12p40 (Figure 4B, *P*<0.001) and for the Th2 cytokine IL-4 (Figure 4B, *P*<0.05). There was a trend for elevated protein levels for the Th3/Tr1 cytokine IL-10, similar to what was observed in most grades of trachoma (Table S1 and data not shown). Among the IL-2 family, the TT/TI cases had elevated levels of IL-2 and IL-15 compared to controls (Figure 4C, *P*<0.05).

In agreement with the decreased Th1 response, CC chemokines MIP-1α and MIP-1β were significantly lower for TT/TI cases compared to controls (Table S1, *P*<0.05 for both). However, CXC chemokines, IL-8 and MCP-1, remain elevated (Figure 4D, *P*<0.05).

### Elevated Th1 and Th2 cytokines and IL-1Ra levels in conjunctival mucosal secretions distinguish trachomatous inflammation from scarring grades of trachoma

**Figure 5:**
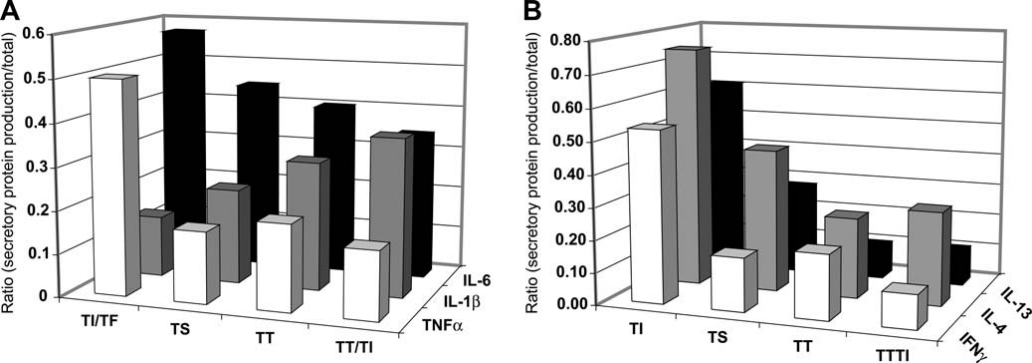
Immuno-regulatory patterns of cytokines and chemokines exist among chronic trachoma cases versus acute TF/TI cases. (A) The TF/TI cases had significantly elevated TNFα mucosal protein production compared to the TT/TI cases (*P*<0.05) and higher IL-6 mucosal production than TS (*P*<0.05). (B) TF/TI cases had significantly elevated production of IL-4 compared to TT/TI cases (*P*<0.01). Further, IL-4 production was detected more frequently for TS cases compared to TT (*P*<0.01) and TT/TI cases (*P*<0.01). Diminished IL-13 production was evident for TT/TI cases compared to TF/TI cases (*P* = 0.005). TF/TI cases had a significantly higher production of IFNγ compared to TT/TI cases (*P*<0.05). Ratios were determined by dividing cases with detectable levels of cytokines and chemokines by the total number of cases in each group. Statistics were based on multiple logistic regression adjusting for *C. trachomatis* infection, age and sex.

Figure 5A shows that TF/TI cases had a higher frequency of TNFα production compared to all other grades, although the difference was only significant between TF/TI and TT/TI (*P*<0.05), likely due to small patient numbers for TS and TT. A reverse trend was apparent for IL-1β, which was supported by the significant reduction in its soluble antagonist receptor (IL-1Ra) concentrations in the TT/TI cases compared with TF/TI and TS cases (data not shown; *P*<0.05 and *P*<0.005 respectively).

Cytokines involved in Th1 and Th2 phenotypes showed a consistent reduction for all grades (TF/TI to TT/TI) (Figure 5B) with TF/TI and TS cases demonstrating more frequent IL-4 production over TT/TI and TT cases (Figure 5B, *P*<0.01, respectively). This correlated with decreased Th2, IL-13, and Th1, IFNγ and IL-12p40, cytokines for TT/TI cases (Figure 5B; *P*<0.05; IL-12p40: OR = 0.80 (0.66–0.97), *P*<0.05 TT/TI versus TS). Among the IL-2 family of cytokines and receptor, decreased IL-2R levels were evident in TT/TI cases compared with TF/TI cases, although these differences were not significant (*P* = 0.082, data not shown). However, these data correlated with elevated IL-2 production in TT/TI compared to TF/TI cases (Table S1), further supporting IL-2 as a risk factor for the development of TT/TI.

A significant reduction in IP-10 levels was found for TT/TI cases compared with TF/TI cases (OR = 0.9980 (0.9961–0.9998), *P*<0.05). Though not significant, a reverse trend was found for MCP-1 levels.

### Elevated protein levels of chemokines, pro-inflammatory and Th3/Tr1 cytokines and lower levels of Th1 and Th2 cytokines in conjunctival mucosal secretions characterized chronic trachoma compared with controls

The effects of chronic trachoma (TS, TT and TT/TI) on inflammatory components were elucidated by grouping these grades and comparing them against their age and sex matched controls. Inflammatory patterns for cases with chronic trachoma compared with controls are shown in Table 2 and Table S2; significantly elevated TNFα, IL-1β and IL-6 concentrations and diminished concentrations of the anti-inflammatory cytokine IL-1Ra were found in chronic trachoma cases compared with controls.

**Table 2:** Association of cytokine and chemokine conjunctival mucosal protein production with chronic scarring trachoma.

Cytokine/Chemokine	Spearman Coefficient^A^	P-value
**Proinflammatory cytokines**		
TNFα	0.168	0.038
IL-1β	0.259	0.001
IL-1Ra	−0.249	0.001
IL-6	0.259	0.001
**Th1/Th2/Th3 cytokines**		
IL-4	−0.165	0.041
IL-13	−0.189	0.019
IL-12p40	−0.212	0.008
IL-10	0.198	0.014
**IL-2 family**		
IL-2	0.249	0.006
IL-15	0.292	0.003
**Chemokines**		
Eotaxin	0.161	0.046
IL-8	0.179	0.026
MCP-1	0.275	0.006
MIG	0.275	0.006
MIP-1α	0.142	0.080

A Data represent association of cytokines/chemokines concentrations with chronic scarring trachoma (TS, TT and TT/TI) compared with age and sex matched controls. Significant differences were determined using Spearman's rank correlation test.

In characterizing host Th1, Th2 and Th3 cytokine levels, IL-4, IL-13 and IL-12p40 were negatively associated with chronic trachoma cases compared with controls (Table 2). However, elevated IL-10 levels were associated with chronic cases (Table 2), further supporting an association with the Th3/Tr1 phenotype and the role of IL-10 as a risk factor for chronic trachoma. Members of the IL-2 cytokine family, IL-2 and IL-15, were also significantly associated with chronic trachoma cases compared with controls (Table 2).

Among the chemokine family, chronic trachoma was significantly associated with elevated levels of Eotaxin, IL-8, MCP-1 and MIG compared with controls (Table 2).

### Influence of *C. trachomatis* infections on cytokine, cytokine receptor and chemokine protein levels in conjunctival mucosal secretions for all grades of trachoma

Table 3 shows the frequency of cytokine, chemokine and cytokine receptor protein concentrations comparing *C. trachomatis* infected and uninfected patients for trachoma cases (all grades) and for controls. TNFα and IL-6 were significantly associated with *C. trachomatis* infections for chronic trachoma cases (Table 3 and S3) with similar patterns for IL-1β, though not significant (Table S3 and S4). This phenotype was not evident among controls (Table 3).

**Table 3:** Association of cytokine and chemokine conjunctival mucosal protein production comparing *C. trachomatis* infections for cases with all grades of trachomatous disease and for controls.

	No Disease		Disease	
Cytokine/Chemokine	Spearman Coefficient^A^	P-value	Spearman Coefficient^A^	P-value
**Proinflammatory cytokines**				
TNFα	−0.043	0.664	0.298	0.002
IL-6	−0.083	0.404	0.206	0.035
**Th1/Th2/Th3 cytokines**				
IFNγ	0.153	0.123	0.188	0.055
IL-2	0.286	0.004	0.107	0.280
IL-15	0.096	0.333	0.304	0.002
IL-10	0.223	0.024	0.233	0.017
**Chemokines**				
Eotaxin	−0.180	0.069	0.072	0.462
MIG	0.138	0.164	0.181	0.065
MIP-1β	−0.115	0.250	0.192	0.050
RANTES	0.012	0.904	0.262	0.007

A Data represent the effect of *C. trachomatis* infection on cytokine and chemokine conjunctival mucosal production for all grades of trachoma (TF/TI, TS, TT and TT/TI) or age and sex matched controls with no disease. Significance between *C. trachomatis* infection and no infection was determined using Spearman's rank correlation test.

Minimal differences were observed for Th1 and Th2 cytokines in the presence or absence of *C. trachomatis* infections (Table S3 and S4). However, elevated IL-10 levels persisted in the presence of *C. trachomatis* infections regardless of trachoma grade (Table 3). Additionally, *C. trachomatis* infections appeared to effect the IL-2 family of cytokines with a significant association of elevated IL-2 concentrations with controls (Table 3); elevated IL-15 was associated with trachoma (Table 3 and Table S3).

In the chemokine family, *C. trachomatis* infection was associated with increased levels of Th1-associated chemokines MIP-1β and RANTES (Table 3). Independent of trachoma grade, MIG was significantly associated with infection (OR = 2.61 (1.25 to 5.45), *P* = 0.011).

## Discussion

While there is some important research on cytokine mRNA expression in association with trachoma, our fundamental knowledge of the host immune response has been limited by a lack of quantitative protein data, especially for those cytokines and chemokines that are post-transcriptionally and –translationally modified, in association with each grade of trachoma. The goal of this study was to validate previous gene expression findings and to characterize novel inflammatory cytokine and chemokine protein concentrations in mucosal conjunctival secretions and how each may influence inflammation for the different grades of trachomatous disease. Additionally, we further characterized immuno-regulatory effects induced by concurrent *C. trachomatis* infection.

Our Nepali study population showed a significant inverse association of *C. trachomatis* infection with age and a direct association with TF/TI cases agreeing with our previous findings and those of others [19],[22],[32].

Proinflammatory cytokines have previously been associated with acute and chronic trachoma [36],[37]. We and others [19],[20] found elevated TNFα levels among TI and chronic trachoma cases and also higher levels when both *C. trachomatis* infection and trachoma were present. Additionally, in our study, elevated IL-1β levels were significantly associated with chronic trachoma cases but not with infection. Previous studies have demonstrated elevated IL-1β mRNA levels during both disease and infection [19],[20]. These differences may be due to post-translational modifications of IL-1β, thus supporting both findings but suggesting the need for quantitative protein studies for confirmation. The previous associations of these cytokines with the induction of scarring-associated proteins, matrix metalloproteins (MMPs) and collagen [19],[20],[21], suggest plausible mechanisms for the development and progression of chronic trachoma. This chronic inflammatory phenotype is further supported in our study by the significantly decreased levels of the anti-inflammatory cytokine IL-1Ra. Recently, Hvid *et al.* characterized the role of IL-1Ra and IL-1α in a human fallopian tube organ culture model [38]. *C. trachomatis* infection resulted in destruction of ciliated and secretory cells within the tubes. However, when co-cultured with IL-1Ra, tissue destruction was minimal while IL-1α exacerbated disease pathology. In trachoma, the prolonged production of TNFα and IL-β together with a reduction in IL-1Ra inhibitory pathways may promote the development of scarring and progression to TT.

The pleiotropic cytokine IL-6 has been associated with chronic trachoma [33]. One previous study evaluated IL-6 gene expression in trachoma but the findings were inconclusive due to detectable mRNA levels in only two patients [19]. We found elevated IL-6 production for all grades of trachoma. Additionally, we demonstrated for the first time a significant association of *C. trachomatis* infection with elevated IL-6 protein levels in both inflammatory and chronic trachoma. Our findings are supported by a previous study demonstrating elevated IL-6 production in the fallopian tubes of macaques after repeated infection with *C. trachomatis* [39]. Murine studies, however, have provided conflicting data. One study showed an increased bacterial burden and mortality associated with pulmonary infection with the mouse pneumonitis strain (MoPn; now referred to as *Chlamydia muridarum*) in IL-6 -/- KO mice [40]. In the mouse genital tract model, however, there was an absence of any pathological or bacterial complications when IL-6 -/- KO mice were infected with *C. muridarum* [41]. Darville *et al.*, found that TLR2 -/- KO mice exhibited decreased levels of IL-6 and oviduct pathology during *C. muridarum* -genital tract infection [42], further supporting IL-6 as a risk factor for chronic trachoma.

Considering the obligate intracellular nature of *C. trachomatis* and previous studies, host cell-mediated immunity (Th1) appears to be critical in eliciting protection against *Chlamydia*-associated diseases. Transcriptional studies have associated elevated Th1 cytokines, IL-12 and IFNγ, mRNA expression with *C. trachomatis* infection for cases with TF/TI [19],[21] but decreased levels with scarring [19]. Wang *et al.* demonstrated elevated endocervical IL-12 protein concentrations in adolescents prior to resolution of their *C. trachomatis* infection [43], further supporting a role for IL-12 in host defense against infection. In our study, we found minimal differences in IL-12p40 concentrations for TF/TI compared with controls but significantly decreased mucosal levels associated with scarring. Additionally, TT/TI cases had lower levels for both Th1 cytokines, IFNγ and IL-12p40, compared to TF/TI. Together, these data suggest that Th1 cytokines are protective immunologic factors against disease progression. While Th2 cytokines are known to be associated with humoral mediated immunity, previous studies have been inconclusive due to undetectable transcriptional levels of these cytokines [19],[21]. In our study, the Th2 cytokines, IL-4 and IL-13, displayed similar patterns to the Th1 cytokines, suggesting that both Th1 and Th2 cytokines may be protective factors against chronic sequelae.

The negative associations of Th1 and Th2 cytokines with chronic trachoma suggest a potential role for Th3/Tr1. The previous categorization of IL-10 as an anti-inflammatory Th2 cytokine has recently been extended given the documented association with T-regulatory (Tregs) cells [44]. Kinjyo *et al.* characterized IL-10 as a Th3/Tr1 cytokine, showing the over production of IL-10 and TGFβ in the absence of a Th2 polarizing gene, SOC3, the suppressor of cytokine signaling [45]. Recently, Faal *et al.* found elevated levels of a T cell regulatory gene, forkhead box 3 (FOXP3), during active trachoma (TF/TI) [22]. This was associated with elevated IL-10 and indoleamine-2, 3-dioxygenase (IDO) mRNA expression levels. IDO levels have been associated with immune tolerance and regulatory pathways [46], which have lead to outgrowth of secondary pneumococcal infections [47]. Additionally, Mark *et al.* demonstrated diminished FOXP3 and IL-10 levels with early clearing of *C. trachomatis* infection in a murine model [48]. In our study, IL-10 was overproduced during all grades of trachoma and with *C. trachomatis* infection. These findings are supported by previous IL-10 studies where elevated mRNA expression was similarly associated with trachoma and infection [20],[21],[22]. In the genital tract, elevated levels of IL-10 have been found in infertile women with documented *C. trachomatis* infections [49] and in macaques that were repeatedly infected with *C. trachomatis* [39]. These collective data support the association of IL-10 with a Th3/Tr1 phenotype and suggest that IL-10 may be a major risk factor for chronic trachoma associated with *C. trachomatis* infection. In contrast, Hvid *et al.* demonstrated reduced *ex vivo* fallopian tube pathology with *C. trachomatis* infection in the presence of excess IL-10, suggesting a protective effect [38] at least during the early stages of infection in the female genital tract. Further investigations are needed to clearly define the role of IL-10 for each trachoma grade.

The involvement of T lymphocytes in the perifollicular area of conjunctival follicles suggests an active involvement of the IL-2 family of cytokines, IL-2, IL-2R, and IL-15, in trachoma. Our IL-2 findings showed an association with chronic trachoma and also agree with a previous study that found an association of IL-2 mRNA expression, although at low levels, with TI and *C. trachomatis* infection [19]. In this study, we demonstrated higher concentrations of IL-15 in cases with scarring and in cases with concurrent *C. trachomatis* infection. IL-15 shares the IL-2Rβ and IL-2Rγ receptors with IL-2, and, therefore, has overlapping bioactivity, which includes the stimulation of proliferation of activated T cells and natural killer (NK) cells [50],[51]. Similar trends in elevated IL-15 protein have been found in the lymph nodes of chronically infected HIV patients [52]. In a murine model, IL-15 KO mice have been shown to have significantly diminished acute and chronic colitis compared to wild-type mice [53], further suggesting that IL-15 production is required to sustain chronic infections. Previous studies have also shown elevated IL-15 mRNA expression in patients with *C. trachomatis*-induced arthritis compared to healthy controls [54]. Immunopathology studies in cynologus monkeys have demonstrated elevated ratios of CD8+ to CD4+ T cell populations in *C. trachomatis* infected naïve compared to orally immunized monkeys [55], suggesting that an over abundance of CD8+ T cells are a risk factor for disease. Recently, IL-15 was linked to extended CD8+ memory T lymphocyte survival rates in mice [56]. IL-15 has also been associated with Treg proliferation [57]. These data support IL-2 and IL-15 as risk factors for chronic trachoma, especially with concurrent *C. trachomatis* infection, which may be associated with abundant CD8+ T lymphocyte populations.

Cellular infiltration resulting in typical trachomatous follicles suggests a major role for chemokines in the progression of disease. However, to our knowledge, there has been no characterization of these proteins in trachoma. Most chlamydial studies that have evaluated chemokines focused on murine and human genital tract infections. In our study, the significant elevation of CXC chemokine IL-8 protein levels for all trachoma grades agrees with previous findings where an abundance of neutrophil populations were present in conjunctival swabs from individuals with trachoma [15]. A chlamydicidal role for neutrophils within the conjunctiva has been demonstrated in one *in vitro* study by Yong *et al.* [58]. However, further studies are needed to characterize these findings *in vivo*. Our findings for IL-8 are also supported by a murine study where the murine form of IL-8, MIP-2, had prolonged production along with neutrophil infiltration and pathology in the genital tract of BALB/c and C3H/HeN mice but not in the more resistant C57BL/6 mouse strain [59]. We found pronounced protein levels for MCP-1 for all grades of trachoma, but lower MIP-1α production for TT/TI cases compared to controls. In addition, there was a trend for elevated MCP-1 in TT/TI compared to TS and TF/TI cases, further associating MCP-1 with chronic grades of disease. These findings are supported by a murine study that found elevated MIP-1α and decreased MCP-1 levels among C57BL/6 mice that had a shorter course of infection [60], suggesting that these patterns of chemokine production are protective. Another murine study exhibited elevated levels of Th1 associated chemokines, RANTES, IP-10 and MIG, with chronic, upper genital tract infections compared to lower genital tract infections for *C. muridarum* [61]. Our study also demonstrated elevated MIG production among chronic trachoma cases with elevated RANTES levels among those who were also infected with *C. trachomatis*.

In conclusion, we characterized the secreted cytokines, chemokines, and cytokine receptor associated with immunopathology for each grade of trachoma and determined the immunomodulating effects of concurrent *C. trachomatis* infection. Our studies are in agreement with others who demonstrated Th1 cytokines as protective and Th3/Tr1 cytokine IL-10 as a possible risk factor for chronic trachoma. Additionally, we reconfirmed previous gene expression studies linking elevated proinflammatory cytokines IL-1β and TNFα with *C. trachomatis* infections, and IL-1β as a strong risk factor for chronic trachoma. Our findings expanded on the IL-1β linkage by demonstrating for the first time, to our knowledge, inverse levels of its antagonist, IL-1Ra, in association with TT, suggesting that IL-Ra is a protective factor against chronic sequelae. We also identified two new risk factors, IL-6 and IL-15, which were associated with chronic trachoma with significantly elevated levels evident with concurrent *C. trachomatis* infection. Currently, we are elucidating signal transduction pathways affiliated with these cytokines and chemokines during *C. trachomatis* infections and their possible inter- and intra-cellular roles during disease. In addition, future vaccine design will likely need to take into consideration the immune responses we have characterized and to ensure a vaccine has the desired protective outcome.

## Supporting Information

Table S1 Frequency of cytokine and chemokine conjunctival mucosal production for different grades of trachoma.
(0.10 MB DOC)

Table S2 Correlation of cytokine and chemokine conjunctival mucosal protein production with chronic scarring trachoma.
(0.03 MB DOC)

Table S3 Association of *C. trachomatis* infection with cytokine and chemokine conjunctival mucosal protein levels for normal, active and chronic trachoma grades.
(0.08 MB DOC)

Table S4 Correlation of cytokine and chemokine conjunctival mucosal protein production with *C. trachomatis* infections for cases with all grades of trachomatous disease and for controls.
(0.05 MB DOC)
