# Supplementary material for: Role of Secreted Conjunctival Mucosal Cytokine and Chemokine Proteins in Different Stages of Trachomatous Disease
Source: PLoS Negl Trop Dis. 2008 Jul 16;2(7):e264. doi: 10.1371/journal.pntd.0000264 (PMC2442224; doi:10.1371/journal.pntd.0000264)
Supplement: Table S1 — Frequency of cytokine and chemokine conjunctival mucosal production for different grades of trachoma. (0.10 MB DOC) [file pntd.0000264.s001.doc]

**Supplemental Table 1.** Frequency of cytokine and chemokine conjunctival mucosal production for different grades of trachoma.

| **Cytokine/**  **Chemokine** | **T0** | **TF/TI^A^** | **T0** | **TS^A^** | **T0** | **TT^A^** | **T0** | **TT/TI^A^** |
| --- | --- | --- | --- | --- | --- | --- | --- | --- |
| TNFα | 35.7 | 64.3* | 5.6 | 55.6** | 40.0 | 40.0 | 44.7 | 44.7 |
| IL-1β | 10.7 | 17.9 | 0.0 | 22.2* | 20.0 | 35.0 | 15.8 | 39.5* |
| IL-1Ra | 96.4 | 100.0 | 100.0 | 100.0 | 100.0 | 95.0 | 100.0 | 94.7 |
| IL-6 | 25.0 | 50.0* | 0.0 | 16.7 | 15.0 | 55.0** | 23.7 | 36.8 |
| Il-4 | 96.4 | 89.3 | 88.9 | 88.9 | 65.0 | 40.0 | 63.2 | 44.7 |
| IL-5 | 3.6 | 0.0 | 0.0 | 11.1 | 0.0 | 0.0 | 2.6 | 0.0 |
| IL-13 | 64.3 | 60.7 | 61.1 | 22.2* | 25.0 | 10.0 | 21.1 | 10.5 |
| IL-12p40 | 96.4 | 92.9 | 100.0 | 100.0 | 95.0 | 75.0 | 100.0 | 71.1** |
| IFNγ | 53.6 | 57.1 | 27.8 | 16.7 | 20.0 | 25.0 | 18.4 | 10.5 |
| IFNα | 7.1 | 3.6 | 0.0 | 5.6 | 0.0 | 10.0 | 2.6 | 0.0 |
| IL-10 | 17.9 | 50.0* | 11.1 | 38.9 | 15.0 | 25.0 | 10.5 | 21.1 |
| IL-2 | 3.6 | 0.0 | 0.0 | 0.0 | 0.0 | 5.0 | 0.0 | 15.8 |
| IL-2R | 92.9 | 92.9 | 83.3 | 77.8 | 70.0 | 60.0 | 76.3 | 65.8 |
| IL-15 | 25.0 | 42.9 | 5.6 | 33.3* | 0.0 | 20.0* | 7.9 | 26.3* |
| IL-7 | 28.6 | 17.9 | 5.6 | 16.7 | 15.0 | 20.0 | 7.9 | 10.5 |
| IL-17 | 14.3 | 3.6 | 11.1 | 5.6 | 0.0 | 0.0 | 2.6 | 0.0 |
| Eotaxin | 21.4 | 32.1 | 16.7 | 38.9 | 25.0 | 25.0 | 31.6 | 34.2 |
| GMCSF | 3.6 | 7.1 | 0.0 | 11.1 | 0.0 | 5.0 | 2.6 | 2.6 |
| IL-8 | 100.0 | 100.0 | 100.0 | 100.0 | 100.0 | 90.0 | 97.4 | 100.0 |
| MCP-1 | 39.3 | 78.6** | 27.8 | 72.2** | 70.0 | 85.0 | 60.5 | 81.6* |
| MIG | 32.1 | 46.4 | 11.1 | 33.3 | 15.0 | 40.0 | 23.7 | 39.5 |
| IP-10 | 100.0 | 100.0 | 94.4 | 83.3 | 80.0 | 100.0 | 92.1 | 81.6 |
| MIP-1α | 60.7 | 57.1 | 55.6 | 72.2 | 95.0 | 95.0 | 94.7 | 76.3* |
| MIP-1β | 75.0 | 75.0 | 22.2 | 100.0** | 75.0 | 45.0 | 73.7 | 50.0* |
| RANTES | 21.4 | 35.7 | 11.1 | 16.7 | 0.0 | 5.0 | 2.6 | 10.5 |

Data represent association of cytokines/chemokines with chronic disease (TS, TT and TT/TI) versus age and sex matched controls. Cytokine/chemokine levels were converted to binomial variables by assigning a 1 to concentrations above background levels and 0 to samples with background levels. Frequency was determined by dividing the number of individuals with detectable cytokine/chemokine protein levels by the total number of patients within the group being analyzed.

^B^Significance between patients within a grade and their age and sex matched controls was denoted as * *P* < 0.05 and ** *P* < 0.01 using multiple logistic regression adjusting for *C. trachomatis*.
