# Supplementary material for: Role of Secreted Conjunctival Mucosal Cytokine and Chemokine Proteins in Different Stages of Trachomatous Disease
Source: PLoS Negl Trop Dis. 2008 Jul 16;2(7):e264. doi: 10.1371/journal.pntd.0000264 (PMC2442224; doi:10.1371/journal.pntd.0000264)
Supplement: Table S2 — Correlation of cytokine and chemokine conjunctival mucosal protein production with chronic scarring trachoma. (0.03 MB DOC) [file pntd.0000264.s002.doc]

**Supplemental Table 2.** Correlation of cytokine and chemokine conjunctival mucosal protein production with chronic scarring trachoma.

| **Cytokine/Chemokine** | **Spearman Coefficient** | **P-value** |
| --- | --- | --- |
|
| **Th1/Th2/Th3 cytokines** | | |
| IFNγ | -0.060 | 0.460 |
| **IL-2 family** | | |
| IL-2R | -0.021 | 0.792 |
| **Chemokines** | | |
| IP-10 | 0.068 | 0.404 |
| MIP-1β | -0.039 | 0.628 |
| RANTES | 0.130 | 0.108 |

Data represent association of cytokines/chemokines concentrations with chronic disease (TS, TT and TT/TI) versus age and sex matched controls. Significant differences were determined using Spearman nonparametric correlation test.
