# Supplementary material for: Role of Secreted Conjunctival Mucosal Cytokine and Chemokine Proteins in Different Stages of Trachomatous Disease
Source: PLoS Negl Trop Dis. 2008 Jul 16;2(7):e264. doi: 10.1371/journal.pntd.0000264 (PMC2442224; doi:10.1371/journal.pntd.0000264)
Supplement: Table S3 — Association of C. trachomatis infection with cytokine and chemokine conjunctival mucosal protein levels for normal, active and chronic trachoma grades. (0.08 MB DOC) [file pntd.0000264.s003.doc]

**Supplemental Table 3.** Association of *C. trachomatis* infection with cytokine and chemokine conjunctival mucosal protein levels for normal, active and chronic trachoma grades.

|  | **T0** | |  | **TI** | | |  | **Chronic** | |  |
| --- | --- | --- | --- | --- | --- | --- | --- | --- | --- | --- |
|  | **no infection** | **CT infection** |  | **no infection** | **CT infection** |  | | **no infection** | **CT infection** |  |
| **Cytokine/ Chemokine** | **Ratio (n=91)** | **Ratio (n=11)** | ***P*-value** | **Ratio (n=14)** | **Ratio (n=14)** | ***P*-value** | | **Ratio (n=63)** | **Ratio (n=13)** | ***P*-value** |
| TNFα | 0.19 | 0.09 | ns | 0.43 | 0.57 | ns | | 0.13 | 0.38 | ns |
| IL-1β | 0.11 | 0.18 | ns | 0.07 | 0.21 | ns | | 0.27 ^A^ | 0.54 | ns |
| IL-1Ra | 1.00 | 0.91 | ns | 1.00 | 1.00 | ns | | 0.95 | 1.00 | ns |
| IL-6 | 0.24 | 0.09 | ns | 0.57 ^A^ | 0.57 | ns | | 0.33 | 0.62 | 0.010 |
| IL-4 | 0.63 | 0.82 | ns | 0.86 | 0.64 | ns | | 0.37 | 0.08 ^A^ | ns |
| IL-13 | 0.37 | 0.64 | ns | 0.64 | 0.57 | ns | | 0.17 ^A^ | 0.00 ^A^ | ns |
| IL-12p40 | 0.99 | 0.91 | ns | 1.00 | 0.86 | ns | | 0.75 ^A^ | 0.92 | ns |
| IFNγ | 0.27 | 0.45 | ns | 0.57 | 0.50 | ns | | 0.14 | 0.15 | ns |
| IL-10 | 0.10 | 0.36 | 0.091 | 0.36 | 0.64 | ns | | 0.21 | 0.31 | ns |
| IL-2 | 0.00 | 0.09 | ns | 0.00 | 0.00 | ns | | 0.06 | 0.23 | ns |
| IL-2R | 0.80 | 0.73 | ns | 1.00 | 0.86 | ns | | 0.68 | 0.54 | ns |
| IL-15 | 0.10 | 0.18 | ns | 0.29 | 0.57 | ns | | 0.22 | 0.54 | 0.014 |
| Eotaxin | 0.24 | 0.00 | ns | 0.21 | 0.29 | ns | | 0.29 | 0.15 | ns |
| IL-8 | 0.98 | 1.00 | ns | 1.00 | 1.00 | ns | | 0.98 | 0.85 | ns |
| MCP-1 | 0.54 | 0.27 | ns | 0.86 ^A^ | 0.71 | ns | | 0.76 | 1.00 | ns |
| MIG | 0.21 | 0.36 | ns | 0.29 | 0.64 | 0.064 | | 0.37 | 0.46 | ns |
| IP-10 | 0.91 | 1.00 | ns | 1.00 | 1.00 | ns | | 0.89 | 0.77 | ns |
| MIP-1α | 0.44 | 0.36 | ns | 0.43 | 0.71 | ns | | 0.54 | 0.69 | ns |
| MIP-1β | 0.67 | 0.45 | ns | 0.43 | 0.71 | ns | | 0.65 | 0.69 | ns |
| RANTES | 0.09 | 0.09 | ns | 0.29 | 0.43 | ns | | 0.08 | 0.23 | ns |

Data represent the effect of *C. trachomatis* infection on cytokine and chemokine conjunctival mucosal production during no disease (T0), acute (TF/TI) and chronic grades of trachoma. Cytokine/chemokine levels were converted to binomial variables by assigning a 1 to concentrations above background levels and 0 to samples with background levels. Frequency was determined by dividing the number of individuals with detectable cytokine/chemokine protein levels by the total number of patients within the group being analyzed.

^A^Significance (*P* < 0.05) between patients of the associated disease grade compared to controls with the same infection status was determined using multiple logistic regression adjusting age and sex.
