# Supplementary material for: Role of Secreted Conjunctival Mucosal Cytokine and Chemokine Proteins in Different Stages of Trachomatous Disease
Source: PLoS Negl Trop Dis. 2008 Jul 16;2(7):e264. doi: 10.1371/journal.pntd.0000264 (PMC2442224; doi:10.1371/journal.pntd.0000264)
Supplement: Table S4 — Correlation of cytokine and chemokine conjunctival mucosal protein production with C. trachomatis infections for cases with all grades of trachomatous disease and for controls. (0.05 MB DOC) [file pntd.0000264.s004.doc]

**Supplemental Table 4.** Correlation of cytokine and chemokine conjunctival mucosal protein production with *C. trachomatis* infections for cases with all grades of trachomatous disease and for controls.

|  | **No Disease** | | **Disease** | | |
| --- | --- | --- | --- | --- | --- |
| **Cytokine/**  **Chemokine** | **Spearman Coefficient** | **P-value** | **Spearman Coefficient** | **P-value** | |
| **Proinflammatory cytokines** | | | | |  |
| IL-1β | 0.059 | 0.554 | 0.126 | 0.202 | |
| IL-1Ra | -0.050 | 0.615 | 0.045 | 0.652 | |
| **Th1/Th2/Th3 cytokines** | | | |  | |
| IL-12p40 | -0.056 | 0.573 | 0.096 | 0.328 | |
| IL-2R | 0.076 | 0.447 | 0.019 | 0.844 | |
| IL-4 | 0.102 | 0.309 | 0.017 | 0.868 | |
| IL-13 | 0.111 | 0.265 | 0.005 | 0.958 | |
| **Chemokines** | | | |  | |
| IL-8 | -0.072 | 0.469 | 0.050 | 0.608 | |
| MCP-1 | -0.140 | 0.160 | 0.057 | 0.564 | |
| IP-10 | 0.148 | 0.135 | 0.086 | 0.384 | |
| MIP-1α | 0.014 | 0.886 | 0.133 | 0.178 | |

Data represent the effect of *C. trachomatis* infection on cytokine and chemokine conjunctival mucosal production for all grades of trachoma (TF/TI, TS, TT and TT/TI) or age and sex matched controls with no disease. Significance between *C. trachomatis* infection and no infection was determined using Spearman’s rank correlation test.
